# Differential Spatial Repositioning of Activated Genes in *Biomphalaria glabrata* Snails Infected with *Schistosoma mansoni*

**DOI:** 10.1371/journal.pntd.0003013

**Published:** 2014-09-11

**Authors:** Halime D. Arican-Goktas, Wannaporn Ittiprasert, Joanna M. Bridger, Matty Knight

**Affiliations:** 1 Lab of Nuclear and Genomic Health, Centre for Cell and Chromosome Biology, Biosciences, School of Health Sciences and Social Care, Brunel University, London, United Kingdom; 2 Biomedical Research Institute, Rockville, Maryland, United States of America; 3 Department of Microbiology, Immunology and Tropical Medicine, School of Medicine and Health Science, George Washington University, Washington, D.C., United States of America; University of Queensland, Australia

## Abstract

Schistosomiasis is an infectious disease infecting mammals as the definitive host and fresh water snails as the intermediate host. Understanding the molecular and biochemical relationship between the causative schistosome parasite and its hosts will be key to understanding and ultimately treating and/or eradicating the disease. There is increasing evidence that pathogens that have co-evolved with their hosts can manipulate their hosts' behaviour at various levels to augment an infection. Bacteria, for example, can induce beneficial chromatin remodelling of the host genome. We have previously shown *in vitro* that *Biomphalaria glabrata* embryonic cells co-cultured with schistosome miracidia display genes changing their nuclear location and becoming up-regulated. This also happens *in vivo* in live intact snails, where early exposure to miracidia also elicits non-random repositioning of genes. We reveal differences in the nuclear repositioning between the response of parasite susceptible snails as compared to resistant snails and with normal or live, attenuated parasites. Interestingly, the stress response gene *heat shock protein (Hsp) 70* is only repositioned and then up-regulated in susceptible snails with the normal parasite. This movement and change in gene expression seems to be controlled by the parasite. Other differences in the behaviour of genes support the view that some genes are responding to tissue damage, for example the *ferritin* genes move and are up-regulated whether the snails are either susceptible or resistant and upon exposure to either normal or attenuated parasite. This is the first time host genome reorganisation has been seen in a parasitic host and only the second time for any pathogen. We believe that the parasite elicits a spatio-epigenetic reorganisation of the host genome to induce favourable gene expression for itself and this might represent a fundamental mechanism present in the human host infected with schistosome cercariae as well as in other host-pathogen relationships.

## Author Summary

Bilharzia is a parasitic disease endemic in many parts of the world. The schistosoma parasite that causes Bilharzia infects humans but uses a fresh water snail as a secondary host. These two organisms have co-evolved together, and as such the parasite will have mechanisms to overcome the host defences. Understanding this delicately balanced relationship is fundamental to controlling or eradicating the disease. We have studied how this parasite can influence how the DNA within the snail behaves. We have shown snail genes have specific locations within the cell nuclei. Further, we have revealed that specific snail genes related to a schistosome infection change to a new non-random nuclear location as they are turned on or up-regulated. We have snail strains that are susceptible or resistant to the infection of parasites and we can also take live parasites and make them unable to complete an infection by irradiating them. In this unique study, we have shown a gene that is involved in stress pathways moves to a new nuclear location and becomes turned on, but only in susceptible snails, infected with fully functional parasite. Our data suggest that this gene is regulated by the parasite, which has control over the host's DNA, so that the gene is moved to an area where it can be actively expressed. We have uncovered a novel mechanism whereby the spatial organization of a host organism is interfered with by a pathogen. This type of control is probably found in other host-pathogen relationships.

## Introduction

The fresh water snail *Biomphalaria glabrata* is an intermediate host for *Schistosoma mansoni* parasite, which causes one of the most prevalent parasitic infections in mammals, known as intestinal schistosomiasis (Bilharzia). The disease is endemic in 77 countries of the tropics and subtropics, and effects over 230 million people yearly whom are infected with *S.mansoni* and other schistosoma species [1]–[2]. Despite the combined use of molluscicides to reduce the snail population along with mass chemotherapy on humans, persistent poverty, inadequate supply of clean water, and the ongoing agricultural and irrigation projects expanding the aquatic habitat of the snail, all limit the effects of control measures taken so far [3]. In view of these challenges long- term control measures for schistosomiasis are being sought. It is hoped that an understanding of the basic biology of the snail – parasite relationship, that can be interfered with, will lead to the development of new control measures that will limit the spread of schistosomiasis by blocking the transmission of the parasite in the snail-host.

*Biomphalaria* snails vary in their compatibility to schistosomes such that some display resistance to infection while others are susceptible [4]. The infection is initiated when a miracidium penetrates a susceptible snail via the head foot region, and subsequently develops into a primary sporocyst. Soon after, however, in a resistant snail the sporocyst is encapsulated by circulating haemocytes, leading to the destruction and elimination of the parasite by a cytotoxic reaction involving free radicals. In contrast, susceptible snails are not able to successfully defend against *S. mansoni* larvae and an infection will develop following miracidial exposure [5]–. Research investigating the transcriptional modulation of genes upon infection has elucidated some of the genetic factors which influence resistance and susceptibility in the snail, such as Fibrinogen Related Proteins (FREPs), heat shock genes, *ferritin*, *actin* [7]–[11] have all been identified. The development of an *in vitro* tissue culture model to support the intramolluscan stages of *S. mansoni* has also aided the investigations into *B. glabrata's* relationship with *S. mansoni* [12]–[14]. Using such a model we have previously utilised the *Biomphalaria glabrata* embryonic (Bge) cell *in vitro* co-culture system to determine spatio-temporal effects on specific genes in the nuclei of Bge cells that have been co-cultured with *S. mansoni* miracidia [15]. Both genes and the chromosome territories that house genes were found to have a non-random radial position within the Bge cell nuclei [15]–[16]. When schistosome miracidia were added to the culture media, large scale nuclear repositioning of specific genes in Bge cells was determined using fluorescence *in situ* hybridisation with specific bacteria artificial chromosomes (BAC) probes and image analysis. The intranuclear movements of genes correlated with the temporal kinetics of gene expression, meaning that genes were repositioned within nuclei with their up-regulation in expression after a signal was transmitted thorough the cell that parasites were in the vicinity. Co-culturing with live attenuated miracidia on the other hand failed to elicit similar gene expression and gene loci repositioning, indicating that normal but not attenuated schistosomes provide stimuli that evoke host responses [15]. This was the first time spatio-epigenetics had been seen in a host challenged by the presence of a parasite and only the second time for any pathogen-host infection. Chromosome territories were seen to change position with a viral infection [17]. These repositioning events are probably initiated by alterations to chromatin modelling which has been shown extensively to be an aspect of host genome control mechanisms in cells infected with bacteria (see for review [18]). Changes in spatial positioning of chromosome territories and/or gene loci are known during physiological processes such as differentiation and development [19]–[20], disease [21]–[27], and cellular proliferation [25], [28]. In order to determine if the gene repositioning seen in the *in vitro* system was a response found also *in vivo* in whole organisms and thus a true parasite induced mechanism it was critical to study this phenomenon in live snails. In the study presented here we utilise the *B. glabrata* fluorescence *in situ* hybridisation technique using snail *ex vivo* cells to determine spatio-temporal effects on three genes in the resistant and susceptible snails after early exposure to *S. mansoni* miracidia. We find the same phenomenon exists *in vivo* and snail genes relocate in snail nuclei soon after exposure to schistosomes, concurrent with the up-regulation of their corresponding transcript. We believe that we are seeing in the intact snail, as previously observed in the Bge- parasite co-culture *in vitro* system, a physical schistosome- specific reorganisation of the host's genome in response to the parasite and by the parasite, in a manner suggesting that early direct influence on a susceptible but not resistant snail's transcriptional activity is controlled by the parasite. This is the first time that this type of pathogen-induced spatio-epigenetics has been observed in an intact whole organism and should be investigated in other co-evolved host:pathogen relationships.

## Results

### Non-random positioning of gene loci occurs in *S. mansoni* exposed intact *Biomphalaria glabrata* snails

**Figure 1:**
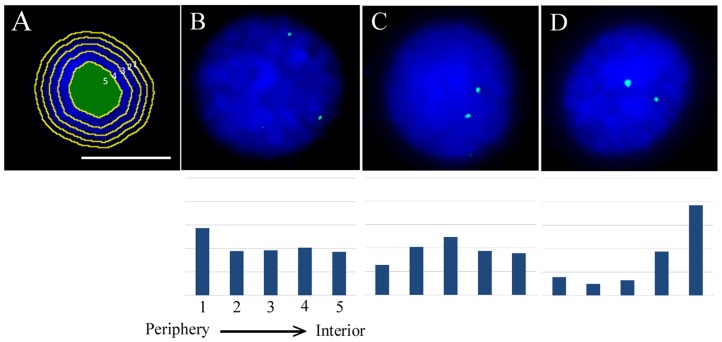
Representative images of the erosion script analysis. Image A displays a composite cartoon of the nucleus where the computer script has outlined the DAPI signal staining DNA (blue) and created five shells of equal area. The script measures the intensity of the fluorescent signals from both the genes (green) and the DAPI and records these. In order to normalise the data, the percentage of gene signal in each shell is divided by the DAPI signal for the corresponding shell. The data can then be plotted as a bar chart. Images B, C, and D are displaying genes having peripheral, intermediate, and internal positions respectively. Scale bar = 10 µm.

**Figure 2:**
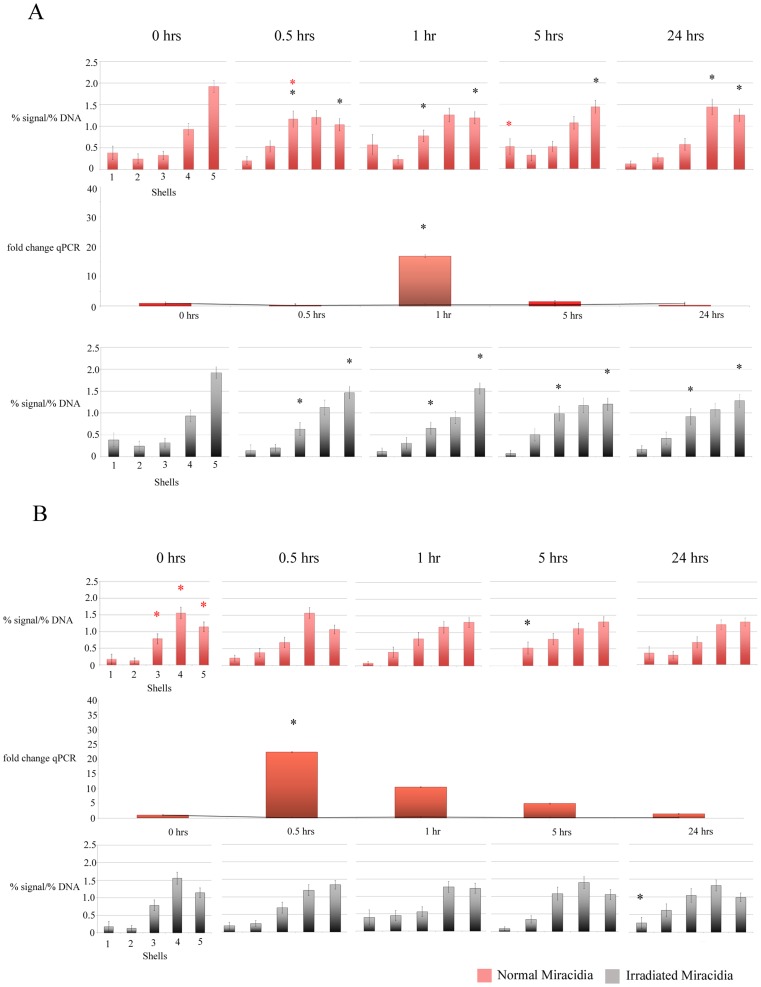
Charts displaying the *in vivo* radial positioning and expression profile of *B. glabrata* actin gene in the interphase nuclei of cells derived from NMRI (A) and BS-90 (B) snail strains, pre and post exposure to *S. mansoni* to normal miracidia (top row of charts, in red) or irradiated attenuated miracidia (bottom row of charts, in grey) over 30 minutes, 2 hours, 5 hours and 24 hours. *B. glabrata* snails were infected with miracidia, dissected, fixed, and subjected to 2-D FISH or RNA was isolated and q-RT-PCR was performed (middle chart). In the NMRI snail strain *actin* is repositioned within interphase nuclei after infection and this is correlated with changes in gene expression (A). No repositioning is observed in the BS-90 snails, however the gene is expressed 30 minutes after infection (B). No repositioning of gene loci or change in expression was observed for *actin* in snails infected with attenuated miracidia. Statistically significant differences, as assessed by two-tailed Student's t-test, between normalized gene signal in each shell of control nuclei compared with infected snail nuclei are indicated by a black asterisk (P<0.05). Student's t-test between normalized gene signal in each shell of NMRI snail nuclei compared with BS90 snail nuclei are indicated by a red asterisk (P<0.05). Error bars = S.E.M.

**Figure 3:**
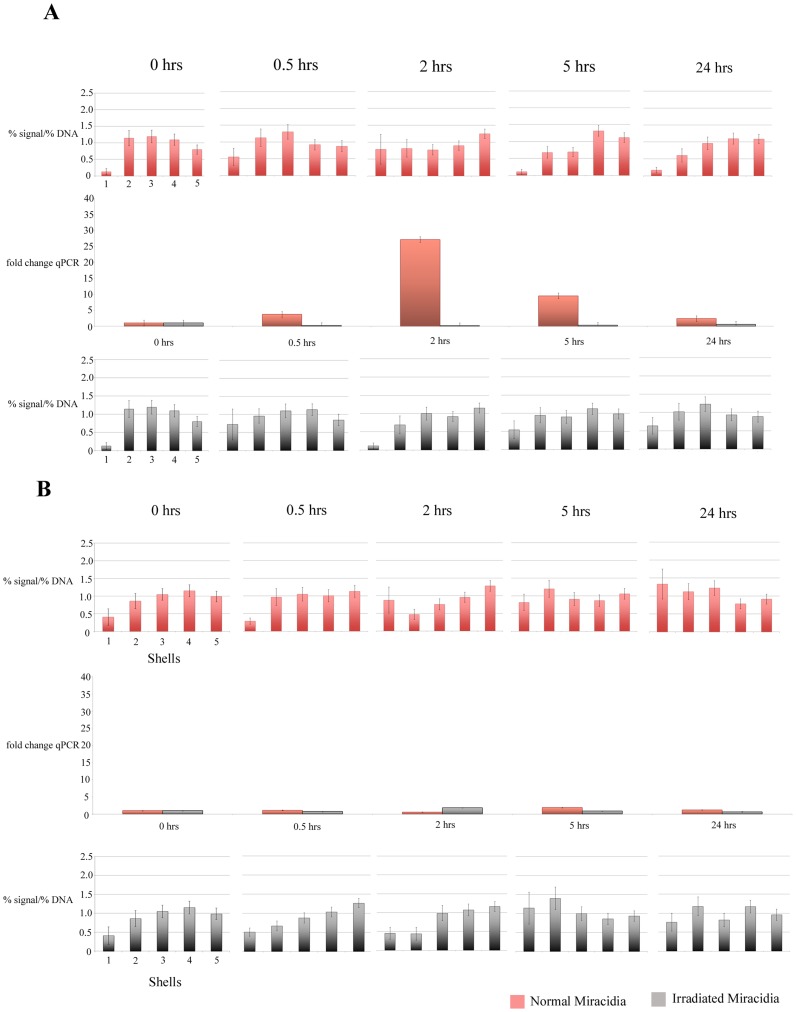
Charts displaying the *in vivo* radial positioning and expression profile of *B. glabrata Hsp* 70 gene in the interphase nuclei of cells derived from NMRI (A) and BS-90 (B) snail strains, pre and post exposure to *S. mansoni* normal miracidia (top row of charts, red) and attenuated irradiated miracidia (bottom row of charts, grey). *B. glabrata* snails were infected with miracidia, dissected, fixed, and subjected to 2-D FISH and RNA was isolated and q-RT-PCR performed (middle chart). In the NMRI strain snail's *Hsp* 70 gene is repositioned after infection from an intermediate position to a more internal position within the nuclei. This repositioning is directly correlated with changes in gene expression 2 hours after infection (A). No repositioning or change in expression is observed in the BS-90 strain snails (B). No evidence for the relocation of the *Hsp* 70 gene loci and no induction of *Hsp* 70 expression were detected by qRT-PCR when the two snail lines were infected with irradiated miracidia. Statistically significant differences, as assessed by two-tailed Student's t-test, between normalized gene signal in each shell of control nuclei compared with infected snail nuclei are indicated by a black asterisk (P<0.05). Student's t-test between normalized gene signal in each shell of NMRI snail nuclei compared with BS90 snail nuclei are indicated by a red asterisk (P<0.05). Error bars = S.E.M.

**Figure 4:**
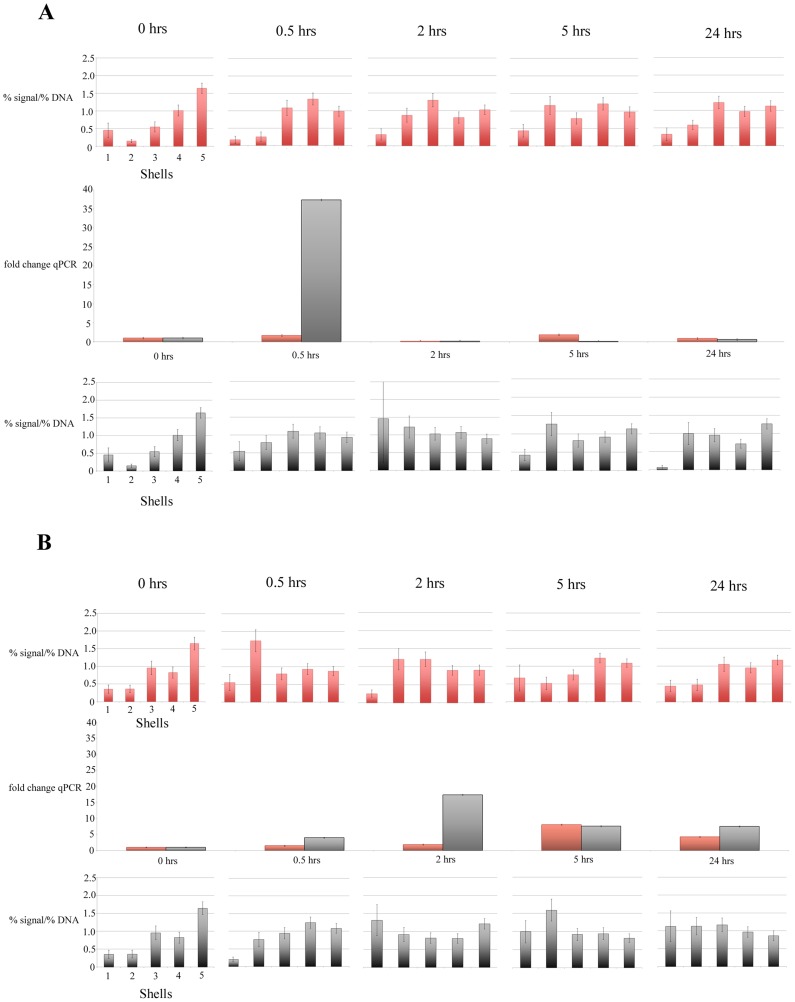
Charts displaying the *in vivo* radial positioning and expression profile of *B. glabrata ferritin* gene in the interphase nuclei of cells derived from NMRI (A) and BS-90 (B) snail strains, pre and post exposure to *S. mansoni* normal miracidia (top row of charts, red) and attenuated irradiated miracidia (bottom row of charts, grey). *B. glabrata* snails were infected with miracidia, dissected, fixed, and subjected to 2-D FISH or RNA was isolated and a q-RT-PCR was performed (middle chart). In the NMRI strain *ferritin* is repositioned in snails infected with both normal and irradiated miracidia. However the gene is up-regulated only in snails infected with irradiated miracidia (A). Repositioning of *ferritin* gene loci is observed in the BS-90 strain snails infected with normal and irradiated miracidia, and this is correlated with up-regulation of its expression (B). Statistically significant differences, as assessed by two-tailed Student's t-test, between normalized gene signal in each shell of control nuclei compared with infected snail nuclei are indicated by a black asterisk (P<0.05). Student's t-test between normalized gene signal in each shell of NMRI snail nuclei compered with BS90 snail nuclei are indicated by a red asterisk (P<0.05). Error bars = S.E.M.

Three genes, *actin*, *ferritin*, and *Hsp 70* that were previously found to be up-regulated after an infection of the snails with *S. mansoni* miracidia [8], [28]–[29] were delineated using fluorescence *in situ* hybridisation (FISH) of labelled BAC probes in interphase nuclei of *B. glabrata* snails from the susceptible (NMRI) and the resistant (BS-90) snail lines. The gene signals can be seen as small concentrated loci in the interphase nuclei and all genes displayed two gene signals, one for each allele (see Figure 1). In order to determine the nuclear position of the gene loci in interphase nuclei, fifty images of nuclei for each gene were captured and their position assessed using a bespoke erosion script analysis (Figure 1A [30]). The script outlines the DNA stain DAPI in collected images of the nuclei and then creates five concentric shells of equal area. The intensity of fluorescent signal from the genes and DAPI is then measured. In order to normalise the 2D data for the original 3-dimensionality of the cells the percentage of gene signal in each shell is divided by the percentage of DAPI signal for the corresponding shell. These data for gene loci are then plotted as a bar chart. The genes are described as having an internal location if the graph is skewed to the right (shell 5), an intermediate location if most of the signals are in shell 3 and towards the nuclear periphery if the graph is skewed to shell 1 (Figure 1). All of the three gene loci displayed non-random radial positioning in interphase nuclei of snail *ex vivo* cells, with *actin* and *ferritin* being located towards the nuclear interior, and heat shock protein (*Hsp 70*) being located at an intermediate position within nuclei. The nuclear location of the genes was similar between the NMRI and BS-90 snail strains (see 0 hours in Figure 2, Figure 3, Figure 4). *Actin* was found in the same nuclear location in the *ex vivo* snail cells as it was in the Bge cells, in the nuclear interior, however, *ferritin* was found in the nuclear interior in the *ex vivo* cells where it was at the nuclear periphery in the Bge cells [15]. This might be due to differences between embryonic cells and adult cells, *in vitro* and *in vivo* cells, or cell type. The embryonic Bge cells appear fibroblast-like but cell type has never been actually identified. The cells from the adult come from the ovotestes and will be a mixture of somatic and germ cells. Only cells with two signals were analysed so that we knew we were assessing adult somatic cells rather than gametes. This ovotestes was selected due to it containing more proliferating cells than other tissues (data not shown) rather than quiescent or senescent cells and we would argue this makes them as a population more responsive. The ovotestes were easier to identify and remove than other tissues ensuring that the population of cells we analysed were similar. We have shown previously, in the pig, that chromosomes are found in similar locations between cells in culture and cells in frozen tissue [31] and so do not feel it is a simply an *in vitro* induced situation. However, Bge cells have been shown to possess an abnormal complement of chromosomes, aneuploidy, which may impact gene positions in these cells [16].

### Gene positioning is altered in *B. glabrata* snails after exposure to *S. mansoni* miracidia and is correlated with gene expression

Infection of *B. glabrata* with *S. mansoni* miracidia modulates host gene expression resulting in the upregulation of certain genes including *actin*, *ferritin*, and *Hsp 70* [8], [28]–[29]. Previously, *actin* and *ferritin* were shown to be non-random repositioned in the *in vitro* co-culture experiments with the parasite and Bge cells [15]. We have now taken a step further to analyse the behaviour of these genes in the susceptible and resistant snail lines after exposure to *S. mansoni* miracidia. Considering that genes and chromosomes can move within a matter of minutes to hours within the nuclear environment [25], [32]–[34], time points were taken at 0, 0.5, 2, 5 and 24 hours after exposing snails to miracidia. To correlate the alteration in the spatial positioning with gene expression, quantitative real time (qRT)-PCR was performed using RNA isolated from parasite-exposed snails for the same time points. In order to determine whether changes observed in gene positioning and gene expression are purely due to the infection process rather than an injury mediated response associated with intra-dermal penetration of the parasite into the snail, nuclei and RNA samples were collected from snails that had been infected with attenuated miracidia.

#### Genes display a differential response with respect to gene repositioning between the susceptible and resistant snail lines

Figure 2 displays the gene positioning of *actin* at different time points in the NMRI and BS-90 snail lines. In the NMRI (susceptible) snails the *actin* gene loci moved from the nuclear interior towards a more intermediate position within the nuclei at 30 min, and subsequently moved back to the nuclear interior after 5 hours. Interestingly however, no major repositioning was observed for the *actin* gene loci in the BS-90 (resistant) snails. The repositioning of *actin* in the susceptible snails correlated with a 16-fold maximum increase in its expression at 2 hours after exposure and 1.5 hours after gene repositioning. The elevation in gene expression was initiated at 30 minutes after exposure, indicating close correlation with spatial repositioning. In the resistant BS-90 snails there was a 22-fold increase in gene expression after only 30 min of infection. Although there was no major movement of the *actin* gene in these snails, the *actin* gene loci were already at a more intermediate nuclear location in the susceptible snails after movement had been induced. T-tests between the position data for 0.5 hours in the susceptible snails and 0 hours in the resistant snails show only shell 2 being statistically different at the 95% confidence interval and between 0.5 hours in the susceptible snails and resistant snails only shell 3 was statistically different at the 95% confidence interval. Although there are some differences we suggest that actin was already in a prime position for transcription in the resistant snails. No repositioning of gene loci or change in gene expression was observed for *actin* in snails infected with attenuated miracidia.

The data for the positioning of the *Hsp 70* gene in timed samples prepared for FISH are displayed in Figure 3. Infection with normal miracidia in the NMRI susceptible snails caused a shift in the position of the gene two hours after exposure, from an intermediate position towards a more internal position within the nuclei, with thegraphs displaying the position of *Hsp70* skewed towards shell 5, the nuclear interior and displaying statistically significant differences for shell 5 at 2 hours and shell 3 at 5 hours. This repositioning event of *Hsp 70* gene loci correlated directly with the upregulation of gene expression as shown by qRT-PCR. In the resistant BS-90 snails however, *Hsp 70* gene loci do not alter their intermediate nuclear position and this is in conjunction with the qRT-PCR data showing no change in gene expression. There are no statistical differences between the positions of the *Hsp70* genes after infection with attenuated parasites when compared to before infection until 24 hours, where there is a statistical difference for shell 1. In the resistant snails there is no change in the location of the *Hsp70* gene nor meaningful increase in gene expression at 2 or 5 hours. Furthermore, there is little gene expression in the resistant snails and there is only a slight difference of gene position with statistical significant differences for shell 1 at 5 and 24 hours only between the resistant and susceptible snails. No evidence for the relocation of the *Hsp 70* gene loci and no induction of *Hsp 70* expression were detected by qRT-PCR when the two snail lines were exposed to attenuated miracidia, indicating that these results reflect a spatio-epigenetic influence from the parasite in susceptible snails. Heat shock proteins have been found as important genes for the parasite to infect its host since in experiments where heat shock protein function is inhibited with a drug geldenamycin the susceptible snail is rendered resistant to parasitic infection [35]. Furthermore, heat shocking the resistant snail makes it susceptible to infection. Revealing the parasite- mediated spatio-epigenetic control over *Hsp 70* expressions in a parasitic infection is a further step in understanding how the presence of heat shock proteins permits an infection.

#### Ferritin may not be involved in the infection process but acts as an injury mediated response gene

The *ferritin* gene behaved differently to the other two genes since it moved in all situations in susceptible, and resistant snails, when exposed to either normal or attenuated parasite. Figure 4 displays the positioning of the *ferritin* gene loci and corresponding gene expression. In the NMRI susceptible snails *ferritin* repositioned from the nuclear interior to a more intermediate position at 30 min post -exposure. Interestingly, *ferritin* gene loci also repositioned at 30 min in these snails exposed instead to attenuated miracida, correlating with a 37-fold increase in the expression of this gene. A similar response was seen in BS-90 resistant snails after exposure to attenuated miracidia. In these snails, *ferritin* repositioned to the nuclear periphery, which once again correlated with its up-regulation as determined by qRT-PCR. There was a dramatic alteration in gene positioning in BS-90 snails after 30 minutes exposure to normal miracidia with the majority of gene loci located in the second shell of the eroded nuclei, more towards the nuclear periphery. This again correlated with an 8-fold increase in the expression of ferritin message at 5 hours. There is only one statistical difference between the ferritin gene movement in the susceptible snails infected with normal parasite as compared to attenuated parasite and that is for shell 2 at 0.5 hours, when we see the correlated upregulation in gene expression. Although a differential temporal response for *ferritin* was observed between the susceptible and the resistant snails, relocation of *ferritin* gene loci and the induction of gene expression in snails infected with attenuated miracidia suggested that this reflected a response to the presence of the miracidia from injury as has been seen in human tissues [36] during the intradermal penetration of the snail host rather than a response to an active infection mechanism elicited by the parasite. Although, it hard to explain why there is a difference in gene expression in the snail when infected with normal or attenuated miracidia it is possible that the attenuated parasites continue to cause an irritation since they do not progress with their life cycle.

## Discussion

Following the initiation of the snail genome project [37], interest in *B. glabrata* has greatly increased, establishing it as the molluscan model organism, especially in host: pathogen studies. While the majority of studies using this model system concentrate on the molecular biology of the snail in order to understand the snail – parasite relationship, only our group examined the genome organisation in this host: pathogen interaction. Thus, in our earlier study [16] where we used an *in vitro* co-culture assay employing Bge cells and schistosome miracidia, we revealed that a profound reproducible reorganisation of the snail genome occurs in response to the presence of normal but not attenuated miracidia [15]. That study was the first to analyse genome behaviour in molluscan cells and to show specific non-random gene repositioning in host cells after exposure to a eukaryotic metazoan parasite. In the study present here we have taken these studies further and observed similar genome reorganisation following infection with the schistosome parasite in intact whole snails. Major gene repositioning events in the interphase nuclei of *B. glabrata* cells were revealed which interestingly differed between parasite resistant and susceptible snail lines. Repositioning of gene loci for *actin*, *ferritin*, and *Hsp 70* all correlated with up-regulation of their expression as determined by qRT-PCR. This fits with an evidence -supported hypothesis in the mammalian genome behaviour field that certain genes, probably those that are either being switched on, or are vastly up-regulated, move to an area of the nucleus where transcription is more active [34] probably to associate with a transcription factory [38]. Due to the lack of whole chromosome painting probes for *B. glabrata* at the present time it is not possible to say if whole chromosomes or genes on chromatin loops have made the transition but the movement is probably initiated by chromatin remodelling with respect to acetylation and mediated thorough nuclear motor proteins. Pathogen chromatin remodelling in the host genome, regulated by a pathogen, has been observed for many bacterial infections [18].

The *actin* gene loci repositioned from the nuclear interior to an intermediate position between the nuclear interior and periphery in the susceptible snails after the infection, but did not alter their position in the resistant snails. The repositioning of gene loci in the susceptible snails correlated with the expression of the gene 1.5 hours after the repositioning event. However in the BS-90 snails there was a rapid response to parasite exposure with a large fold increase in the expression of *actin* after 30 min. Although in the BS-90 snails no repositioning was observed for the gene loci at any of the 0.5, 2, 5, and 24 hour time points, alterations in the organisation of interphase nuclei can be a very rapid process of less than 15 mins after a change in status in a cell [25]. Therefore it may well be that the gene was repositioned and moved back to the nuclear interior within 30 min. On the other hand the *actin* gene loci in the BS-90 cells before infection were in a similar nuclear position to the *actin* loci in NMRI cells after exposure which could mean that this gene does not need to move far to reach an amenable transcription factory. Actin protein is a major constituent of the cytoskeleton and is involved in a number of cellular processes [39]. Increase in the expression of *actin* has been reported in plant- fungal pathogen interactions [40]. Thus the rapid increase in expression observed in the BS-90 snails could be due to rearrangements in the host cytoskeleton that the NMRI snails being susceptible fail to respond as efficiently. The possibility that the actin gene is already in place within nuclei to respond more rapidly to an infection could be linked to what makes BS-90 resistant to an infection.

We feel one of the most interesting findings in this study is that *Hsp70* relocation and expression are tightly temporally correlated at 2 and 5 hours in susceptible snails which is shown to be significant using t-tests. This relocation and expression is only found in susceptible snails with normal but not attenuated parasite. We know that the heat shock proteins are intimately involved in a positive outcome for the parasite since exposing the BS-90 snail to heat changes it from a resistant snail to becoming susceptible to parasite infection [35] and blocking the function of the *Hsp 90* genes makes the susceptible snail NIMR resistant. We also know that a rise in temperature in snails increases *Hsp 70* gene expression and this correlates with the *Hsp 70* gene loci changing their location (H. Arican-Goktas, M. Town, CE Eskiw, M. Knight and JM Bridger unpublished data). It is therefore possible that a stress response that includes *Hsp 70* expression permits an infection and this is controlled through an epigenetic pathway such as spatial positioning and/or transcriptional regulation of the gene. Evidence for this is found in the resistant BS-90 snails where we find neither *Hsp 70* gene movement nor gene expression similar to the susceptible NIMR snails when exposed to attenuated parasites (Figure 3).

In this study we have shown that relocation of genes correlated with change in their transcriptional status and for the first time we have provided evidence for the movement of a gene (*actin*) prior to an alteration to its transcriptional activity, occurring 1.5 hours after relocation. Thus, we have some evidence to add to the debate on what comes first; gene expression or relocation. Although, in order for a gene to be moved to a new location there needs to be signal and this is probably epigenetic via chromatin modification followed by a nuclear motor activity. The snail genome does carry marks of CpG island methylation [41] and histone modifications [42] and detailed analysis of many of the chromatin modifications in these snail genes before and after an infection will be the basis of a future study from our laboratories. Further, it is not unlikely that the parasite could have co-evolved to epigenetically control host gene expression to aid their infection and survival. This is already known to happen in host/bacterial relationships where the bacteria elicit chromatin remodelling events i.e. histone modification, DNA methylation and affect transcription and processing of RNA [18]. Thus, there is no reason why in the more complex co-evolved relationship of schistosome with the snail- host that the parasite would not have also developed such regulatory control over both its hosts, snail and human. Indeed, it has recently been shown that schistosomes already use epigenetic control for gene expression that permits the parasite to invade specific strains of host snail [43]. Furthermore, epigenetic control involving cytosine methylation has been shown to play a role in the parasite life cycle, including the egg laying process [44]. These, novel data on gene movement in the snail host mediated by a pathogen that is far more complex than either viral or bacterial infections where spatio- epigenetics has been shown to also occur should help uncover fundamental elements of genome behaviour permitting infection in other host: pathogen systems, most importantly, in the human host and schistosome parasite relationship.

## Materials and Methods

### *Biomphalaria glabrata* stocks and parasite exposure

Adult *B. glabrata* snails from the susceptible line (NMRI) and the resistant line (BS-90) were incubated overnight in sterile distilled water containing 100 µg/mL ampicillin at room temperature in an attempt to reduce contaminating resident bacteria in the snails. Individual snails were exposed to either attenuated or normal miracidia (10 miracidia per snail) at five different time points (0, 0.5, 2, 5, 24 hours) post-infection. Attenuated miracidia were obtained by irradiation (20 Krad) as described by Ittiprasert et al 2009. Following parasite exposure the snail ovotestes were dissected and incubated in hypotonic potassium chloride solution (0.05 M) for 30 min, during which time the tissues were macerated with a needle to obtain single cells. The cells were then centrifuged at 163 g for 5 min and fixed with methanol and acetic acid (3∶1 v/v) for 15 min at room temperature. The fixed cells were washed five times with methanol and acetic acid (3∶1 v/v) and stored at 4°C until further use. The cells of the ovotestes were chosen since they contain more proliferating rather than quiescent or senescent cells than other areas of the snail and so maybe as a population be more responsive to an infection. The ovotestes are also easier to isolate than other tissues ensuring that similar cells are being compared. Cells were dropped onto wet glass microscope slides to prepare interphase nuclei samples for fluorescence *in situ* hybridisation (FISH).

### Quantitative real time-PCR

Total RNA from the whole body of *B. glabrata* adult snails (∼8–12 mm shell diameter) either unexposed (0 min) or exposed was extracted individually by RNAzol RT (Molecular Research Center, Inc.) according to the manufacturer's manual. With this reagent the contaminated residual DNA was eliminated as previously described [45]. Quantitative RT-PCR was performed by one-step reaction using Brilliant II SYBR green QPCR master mix (Stratagene, Agilent). The validation method (ABI manufacturer's instructions) and melting curve was optimized by using different input RNA (four different sample dilutions) containing *ferritin*, *actin*, *Hsp 70* and *myoglobin*, a house keeping gene [45] to confirm that the amplification efficiencies of both genes were equal and contained a single peak at the expected temperature to indicate target- specific amplification (data not shown). We chose to use myoglobin as the housekeeping because it an abundant transcript that is constitutively and uniformly expressed in all tissues of *B. glabrata* irrespective of their susceptibility to the parasite. While others prefer actin, in our hands, this transcript is differentially regulated upon infection depending on whether or not snails are resistant or susceptible to the parasite. Furthermore actin is a nuclear motor protein and maybe used in moving chromatin around after an infection and is not used as a control gene in Q-PCR in studies interested in genome reorganization. Using custom designed primers that were synthesized by Operon Biotechnologies, the gene specific primers (GSPs) for Q-RT-PCR, showing no cross hybridization to *S. mansoni*, were designed using Primer-Blast algorithm in GenBank (T_a_ = 58°C). Twenty-five microliters of each RT-PCR mixture contained 100 ng total RNA, 12.5 µl Brilliant II SYBR green PCR master mix, 150 nM of each gene specific primers (GSPs): *actin*, ferritin and *Hsp 70* and 1 µl of blocking reverse transcriptase. Individual snail RNA samples were run in triplicate, and all assays contained a no template negative control to rule out non- specific amplification from contamination in the buffers. The Q-RT-PCR data were normalized using myoglobin (Mb) as housekeeping gene as previously described (7). The sequences of each primer are: 1) *actin* (Acc. no. CO501282); *actin*-F 5′-GTCTCCCACACTGTACCTATC-3′, *actin*-R 5′-CGGTCTGCATCTCGTTTTC-3′, 2) ferritin (Acc. no. AW739595); ferritin –F 5′-GGAGGAGAGAGAACATGC-3′, ferritin-R 5′-CACCAATCTGCTTGATGGAC-3′, 3) *Hsp* 70 (Acc. no. L44127); *Hsp* 70-F 5′-AGGCGTCGACATTCAGGTCTA-3′, *Hsp* 70-R 5′-TGGTGATGTTGTTGGTTTTACCA-3′ and 4) myoglobin (Mb; Acc. no. U89283); Mb-F 5′-GATGTTCGCCAATGTTCCC-3′ and Mb-R 5′-AGCGATCAAGTTTCCCCAG-3′ [35], [45]. The transcript levels of *ferritin*, *actin* and *Hsp* 70 during different time points following exposure were normalized relative to myoglobin expression as mentioned above. Fold change of gene expression were calculated by the comparative Ct method with the formula indicated below [46]:
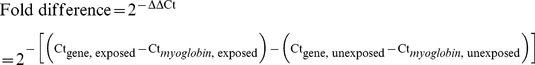



The gene fold change difference between unexposed and exposed snail groups was compared by Student's t-test, with *P*-values <0.05 (N = 10 for each group) showing differentially expressed transcripts between the two groups that were significant. In these experiments, we performed Q-RT-PCR, in triplicate, from RNA samples isolated from 10 individual snails per time point for each group with 2 biological replicates.

### Fluorescence *in situ* Hybridisation

DNA probes for the *B. glabrata* genes for fluorescence *in situ* hybridisation (FISH) were derived from clones of *B. glabrata* bacterial artificial chromosome (BAC) libraries (BB02 stocks), containing *actin*, *ferritin*, and *Hsp 70* coding sequences [47]. FISH was performed as previously described with BAC DNA being labelled with biotin using nick translation and visualised via streptavidin conjugated to cyanine 3. After denaturation of the sample and probe, slides were placed at 37°C overnight to facilitate hybridisation [15], [48].

### Image analysis

Nuclei stained with 4′,6-diamidino-2-phenylindole (DAPI) were observed using the Olympus BX41 fluorescence microscope and UPlanFLN 100x/1.30 oil immersion objective. Digital images were captured using a grayscale digital camera (Digital Scientific UK) and the Smart Capture 3 software (Digital Scientific UK). Fifty images of nuclei for each gene (*actin, ferritin, Hsp 70*) were captured and the position of the gene assessed using the erosion script analysis [30] (Fig. 1) as described previously [15]. The computer analysis script uses the grayscale images to assess the position and intensity of the DNA and FISH signals. Statistical analyses were performed by using unpaired, two-tailed Student's *t* test.

### List of gene accession numbers

*B. glabrata* actin Acc.no. CO501282, *B. glabrata* ferritin Acc. No. AW73959, *B. glabrata Hsp 70* Acc. No. L44127, *B. glabrata* myoglobin Acc. No. U89283.
